# Wealth and health: the neglected economic determinant

**DOI:** 10.1016/j.lanepe.2024.101138

**Published:** 2024-11-16

**Authors:** Rebeka Balogh, S Vittal Katikireddi

**Affiliations:** MRC/CSO Social & Public Health Sciences Unit, University of Glasgow, UK

Wealth has received relatively little attention as an economic determinant of health compared to income.^1^ This scarcity of empirical research is particularly striking given wealth inequalities exceed those of income,^2^ and a recognition that wealth better captures long-term (dis)advantage over the life course.^3^ This evidence gap is likely driven by difficulties in obtaining detailed information on assets and liabilities that make up wealth.^1^^,^^3^

Register data in countries with wealth taxation (where collecting information on wealth was mandated) can help fill the gap in better understanding links with health outcomes.^4^ In this issue of *the Lancet Regional Health—Europe*, Gugushvili and Wiborg drew on nationwide Norwegian register data to assess links between wealth measured at age 37–38 and mortality up to age 62 in the general population, and then used sibling and twin comparisons to better account for shared family characteristics.^5^ Their analysis showed that the mortality rate of men at the lower end of the gross wealth distribution was over twice as high than that of men at the top of the wealth distribution (hazard ratio (HR) 2.39; 95% confidence interval (CI) 2.02–2.83), with the mortality difference only slightly lower for women (HR 1.74; 95% CI 1.39–2.16). Mortality inequalities were particularly pronounced in the lower half of the wealth distribution.

The association between wealth and mortality was robust to adjustment for individuals’ observed characteristics, such as debt and earnings, and persisted even when accounting for unobserved characteristics shared by siblings or when restricting the analyses to twins only. This study design was therefore able to highlight that residual confounding from various environmental and genetic factors did not fully explain the links between wealth and mortality. A further notable contribution of the study was conceptual in nature. The authors considered gross wealth, the sum of different assets without subtracting any debts, as their primary indicator while also accounting for debt levels independently. Net wealth, whereby any debts are deducted from assets, can be misleading they argued. Different levels of gross wealth and debt (but comparable levels of net wealth as a result of the two offsetting each other) can be associated with different social positions, and, subsequently, mortality rates. The study’s empirical testing indeed showed that the multidimensionality of wealth should not be ignored in epidemiological analyses going forward. The gender dimension should not be either: associations between wealth and subsequent mortality were more pronounced for men than women.

The paper has, importantly considered the multidimensionality of wealth (and debt) and even looked at some of its components (finance and real capital) separately in relation to mortality. Future research will hopefully go further by investigating more in detail how the differential make-up of wealth affects mortality and other health outcomes. In the United Kingdom, for instance, physical assets make up the majority of wealth for those at the lower end of the distribution, while for higher levels of wealth, private pension wealth is the most notable element.^6^

The authors largely relied on a particular measure for the wealth indicator whereby the focus was on where individuals stood in the wealth distribution rather than their absolute levels of wealth. Such rank-based measures are not able to examine if there are thresholds that may be protective in terms of mortality rate.^7^ Similarly, while the authors made a clear case why net wealth (assets minus debt) should not be prioritised as a wealth indicator, there is a particular case where net wealth may however warrant an examination: negative wealth (where one’s debt exceeds assets).^8^

Prior research^4^ and Gugushvili and Wiborg’s paper^5^ have attempted to assess whether wealth predicts mortality independently of income/earnings. The two, however, are linked: earnings contribute to wealth and wealth may influence income (e.g., as dividends, interest, or altering decisions about paid work).^9^ This complex interplay between wealth and income along with other social determinants of health needs to be examined, and preferably across countries with differing welfare systems where the effects of wealth on health may operate differently.^4^ Elucidating the causal effect of wealth on health could help us understand how a potential intervention, such introducing or changing wealth taxes, would affect population health and health inequalities. Simulations are starting to emerge^10^ and we hope more will follow.

Gugushvili and Wiborg’s work^5^ is a welcome contribution to the evidence base around wealth as an economic determinant of health and we hope it encourages future work to further unravel its complexity with the ultimate aim of producing actionable evidence to inform healthy economic policy.

## Contributors

RB wrote the first draft based on ideas jointly developed by RB and SVK, with both authors editing and approving the final version.

## Declaration of interests

We declare no competing interests, except for the funding acknowledged.
